# Potential Risk of Cognitive Impairment Due to Irradiation of Neural Structures in Locally Advanced Nasopharyngeal Cancer Treated by Curative Radiotherapy

**DOI:** 10.3390/medicina61050810

**Published:** 2025-04-27

**Authors:** Camil Ciprian Mireștean, Călin Gheorghe Buzea, Alexandru Dumitru Zară, Roxana Irina Iancu, Dragoș Petru Teodor Iancu

**Affiliations:** 1 Regional Institute of Oncology, 700483 Iaşi, Romania; mc3313@yahoo.com (C.C.M.); zara.alexandru@gmail.com (A.D.Z.); dt_iancu@yahoo.com (D.P.T.I.); 2 “Prof. Dr. N. Oblu” Emergency Clinic Hospital, 700309 Iași, Romania; calinb2003@yahoo.com; 3 Oral Pathology Department, Faculty of Dental Medicine, “Gr. T. Popa” University of Medicine and Pharmacy, 700115 Iaşi, Romania; 4 “St. Spiridon” Emergency Hospital, 700111 Iaşi, Romania; 5 Oncology and Radiotherapy Department, Faculty of Medicine, “Gr. T. Popa” University of Medicine and Pharmacy, 700115 Iaşi, Romania

**Keywords:** nasopharyngeal cancer, radiotherapy, IMRT, VMAT, 3D-CRT, organs at risk, OARs, temporal lobe, hippocampus, cognitive impairment, radionecrosis

## Abstract

*Background and Objectives*: Brain radionecrosis is an under-recognized but potentially life-altering late complication of radiotherapy in patients with locally advanced nasopharyngeal cancer. Temporal lobe radionecrosis and high-dose exposure to the hippocampus are strongly associated with cognitive decline and radiation-induced dementia, negatively impacting patients’ long-term quality of life (QoL). This study aimed to evaluate and compare radiation dose distributions to critical brain structures across three radiotherapy techniques—3D conformal radiotherapy (3D-CRT), intensity-modulated radiotherapy (IMRT), and volumetric-modulated arc therapy (VMAT)—in order to assess potential neurocognitive risks and support hippocampal-sparing protocols. *Materials and Methods*: Ten patients previously treated with 3D-CRT were retrospectively replanned using IMRT and VMAT techniques on the Eclipse v13.3 (VARIAN) planning system. Bilateral hippocampi and temporal lobes were delineated as organs at risk (OARs) according to the RTOG atlas, and dosimetric parameters including D_max, D_mean, and D_min were recorded. V7.3 values were evaluated for hippocampal avoidance regions. *Results*: While IMRT and VMAT provided improved target volume coverage and reduced high-dose exposure to many standard OARs, both techniques were associated with increased D_mean and D_min to the hippocampus and temporal lobes compared to 3D-CRT. The highest D_max values to the temporal lobes were observed in 3D-CRT plans, indicating a potential risk of radionecrosis. VMAT plans showed hippocampal mean doses exceeding 10 Gy in some cases, with V7.3 > 40%, breaching established neurocognitive risk thresholds. *Conclusions*: These findings support the routine delineation of the hippocampus and temporal lobes as OARs in radiotherapy planning for nasopharyngeal cancer. The implementation of hippocampal-sparing strategies, particularly in IMRT and VMAT, is recommended to reduce the risk of radiation-induced cognitive toxicity and preserve long-term QoL in survivors.

## 1. Introduction

Radiotherapy is one of the cornerstones of treatment for nasopharyngeal cancer, typically administered in association with cisplatin-based chemotherapy. This combined approach has significantly improved locoregional control and overall survival in patients with locally advanced disease. The development of modern radiation techniques has substantially reduced the risk of treatment-related toxicities, allowing more precise targeting of tumor volumes and better protection of surrounding healthy structures. However, despite these technological advancements, cognitive impairment associated with irradiation of certain brain structures remains one of the most serious and underappreciated late complications.

Two key anatomical structures, the temporal lobes and the hippocampus, play a decisive role in cognitive processes such as memory formation, learning, and executive function. The proximity of these structures to the skull base makes them particularly vulnerable in radiotherapy for nasopharyngeal cancer. Irradiation of the hippocampal region has been shown to lead to neurocognitive effects in patients treated for primary cerebral tumors and intracranial metastases. However, similar complications are increasingly being reported in patients who undergo curative-intent radiotherapy for locally advanced nasopharyngeal cancer, where radiation fields often extend close to or include portions of these critical brain structures [1].

Radionecrosis of the temporal lobes, secondary to high-dose irradiation of the skull base, represents one of the most severe late complications in these patients. Clinically, temporal lobe radionecrosis can manifest as memory deficits, cognitive slowing, mood changes, and, in severe cases, progressive dementia [1]. Radiographic studies confirm these changes, often appearing months or even years after treatment. Such complications are especially concerning given that many patients with nasopharyngeal cancer are long-term survivors.

The replacement of conventional radiotherapy with three-dimensional conformal radiotherapy (3D-CRT) and, subsequently, with intensity-modulated radiotherapy (IMRT) for head and neck cancers has led to significant reductions in late-onset toxicity [2,3]. These advanced techniques offer improved dose conformity around the tumor target volumes and allow for better sparing of critical structures. IMRT, in particular, has been associated with lower incidences of severe side-effects, such as grade 3 or higher xerostomia and mucositis, while maintaining excellent tumor control rates [2,3].

However, the implementation of IMRT and other highly conformal techniques comes with its own set of challenges. While these techniques reduce high-dose exposure to certain areas, they may inadvertently increase the volume of normal tissues receiving low-to-moderate doses of radiation, known as the “low-dose bath”. This effect can potentially result in subclinical or delayed toxicities that accumulate over time, affecting cognitive performance and quality of life [4,5]. The hippocampus and temporal lobes, if not specifically delineated as organs at risk, may be exposed to unintended radiation doses that can contribute to long-term cognitive decline [6,7].

Additionally, cumulative neurotoxicity arising from combined chemotherapy and radiotherapy further exacerbates the risk of cognitive impairment. This phenomenon, commonly referred to as “chemo-brain”, includes symptoms such as memory loss, decreased concentration, and difficulty in processing information. Chemotherapy-induced neuroinflammation, oxidative stress, and neuronal damage contribute to these impairments, even in the absence of direct blood–brain barrier penetration by cytotoxic agents [8,9]. The synergistic effect of chemotherapy and radiotherapy on cognitive decline underscores the need for careful treatment planning and optimization to mitigate these risks.

Furthermore, tumors involving the skull base, such as glioblastomas, present similar challenges in radiotherapy planning, where the balance between tumor control and the preservation of neurocognitive function is crucial. Understanding these risks across different tumor types highlights the necessity of hippocampal sparing and precise dose management, not only in intracranial tumors but also in extracranial malignancies, such as nasopharyngeal cancer [10,11].

Recent international recommendations—including those from the Radiation Therapy Oncology Group (RTOG), QUANTEC (Quantitative Analyses of Normal Tissue Effects in the Clinic), and NRG Oncology—have highlighted the importance of delineating neural structures such as the hippocampus and temporal lobes as organs at risk. Dose constraints, such as limiting the dose to <7.3 Gy for no more than 40% of the bilateral hippocampus (V7.3 < 40%), have been proposed based on evidence correlating dose exposure with cognitive outcomes in patients undergoing whole-brain radiotherapy [7,12,13]. While originally developed for patients with intracranial disease, these constraints are increasingly considered relevant for extracranial cases like nasopharyngeal cancer, where long-term cognitive outcomes are similarly impacted.

In conclusion, although modern radiotherapy techniques have reduced many treatment-related toxicities, the risk of radiation-induced damage to the hippocampus and temporal lobes remains a critical concern. Cognitive decline associated with radiation-induced injury in these regions has a significant impact on long-term quality of life. Therefore, identifying these structures as organs at risk and applying strict dose constraints should become standard practice in the radiotherapy planning process for nasopharyngeal cancer. This study aims to evaluate the dosimetric impact of different radiotherapy techniques on the hippocampus and temporal lobes in patients treated with curative intent for locally advanced nasopharyngeal cancer, emphasizing the importance of incorporating hippocampal-sparing strategies into clinical practice.

### Structure of the Paper

This paper is organized as follows:

**Materials and Methods**: Describes the patient cohort, radiotherapy techniques used (3D-CRT, IMRT, VMAT), imaging and contouring protocols, and dosimetric analysis based on Eclipse planning software.

**Results**: Presents a comparative analysis of radiation doses delivered to the hippocampus and temporal lobes across all planning techniques, highlighting key parameters (D_mean, D_max, D_min, and V7.3).

**Discussion**: Interprets the clinical significance of the findings in the context of the current literature, discusses implications for long-term cognitive outcomes, and supports the implementation of hippocampal and temporal lobe-sparing strategies.

**Conclusions**: Summarizes the importance of considering cognitive function preservation as part of standard planning in radiotherapy for nasopharyngeal cancer and recommends future directions.

## 2. Materials and Methods

### 2.1. Study Workflow Overview

**Figure 1:**
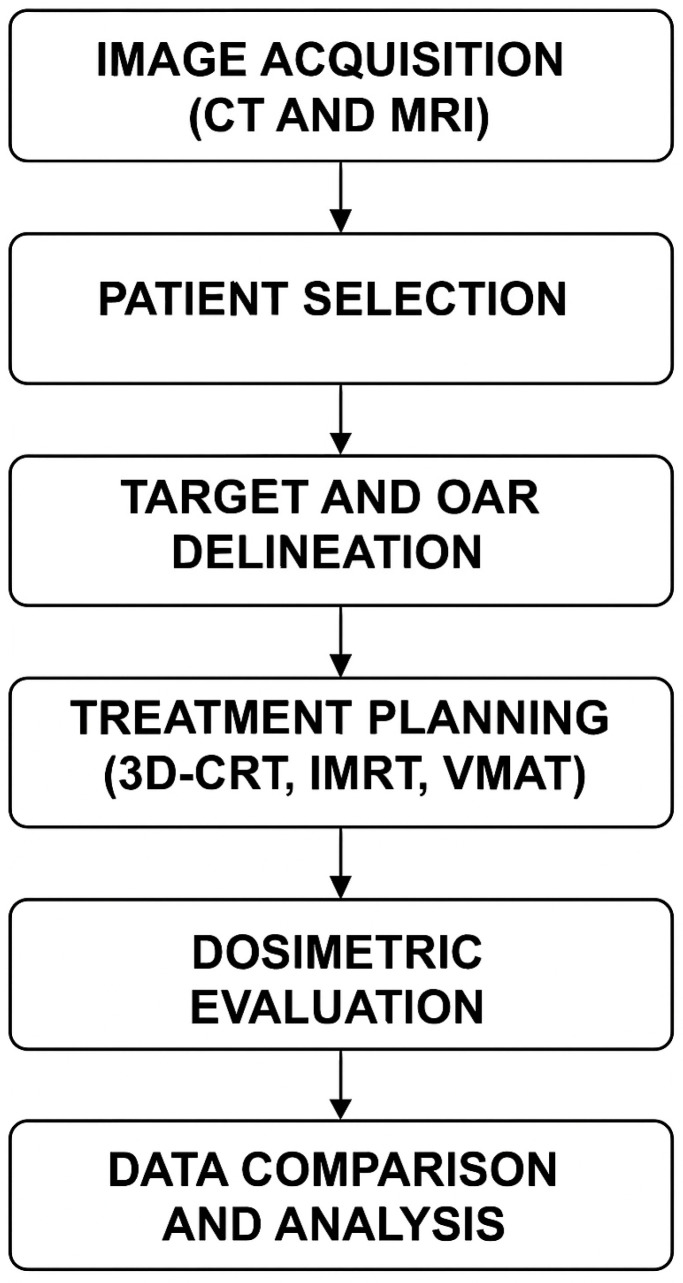
Workflow of the study methodology: from image acquisition (CT and MRI) and patient selection to delineation, treatment planning with three techniques (3D-CRT, IMRT, VMAT), dosimetric evaluation, and final comparative analysis.

The overall workflow for this retrospective planning study is outlined in Figure 1, summarizing the key steps from imaging to data analysis.

This retrospective planning study was conducted on a cohort of ten patients diagnosed with locally advanced, non-metastatic nasopharyngeal carcinoma who received curative-intent chemoradiotherapy between 2012 and 2014. All patients were initially treated using three-dimensional conformal radiotherapy (3D-CRT), and their treatment plans were subsequently re-evaluated using modern planning techniques, including intensity-modulated radiotherapy (IMRT) and volumetric-modulated arc therapy (VMAT), for comparative dosimetric analysis.

### 2.2. Imaging and Delineation Protocol

Target volumes and organs at risk (OARs), including the spinal cord, brain, brainstem, parotid glands, optic nerves, optic chiasm, and lenses, were delineated on computed tomography (CT) simulation scans acquired from the vertex to the carina, with a slice thickness of 3 mm. To ensure accurate gross tumor volume (GTV) delineation, a rigid registration algorithm was used to fuse contrast-enhanced diagnostic magnetic resonance imaging (MRI) or computed tomography (CT) scans with the simulation CT dataset. Endoscopic findings and clinical data were also integrated into the target volume definition process.

Clinical target volumes (CTVs) were defined by applying 5 mm expansions to the GTVs, adjusted for anatomical barriers and patterns of microscopic spread. Planning target volumes (PTVs) were created by applying an additional 5 mm isotropic margin to the CTVs, accounting for setup errors and organ motion, in line with established radiotherapy planning standards [14].

### 2.3. Treatment Planning and Dose Prescription

The original treatments were delivered using a sequential boost technique in three phases. The total dose prescribed to the primary tumor volume was 70 Gy, delivered in 35 fractions. Cervical lymph nodes received a total dose of 66 Gy in 33 fractions, and the supraclavicular lymphatic regions were treated with 50 Gy in 25 fractions. All treatments were performed using 3D-CRT plans.

For each patient, two additional comparative plans were retrospectively generated using the Eclipse v13.3 (VARIAN Medical Systems) treatment planning system. The IMRT plans employed a 7–9 fixed field technique, while VMAT plans used dual-arc configurations. All plans were normalized to ensure that at least 95% of the PTV received 95% of the prescribed dose (V95 ≥ 95%).

### 2.4. OAR Delineation and Dose Evaluation

In addition to standard OARs, bilateral hippocampi and temporal lobes were delineated in accordance with the Radiation Therapy Oncology Group (RTOG) atlas, based on fused T1-weighted MRI and planning CT images [15]. The hippocampal avoidance region was created using a 3 mm isotropic expansion around the hippocampus, following the methodology proposed by Gondi et al. [16].

Dosimetric parameters evaluated for the hippocampi, hippocampal avoidance area, and temporal lobes included:

**D_max** (maximum dose),

**D_mean** (mean dose),

**D_min** (minimum dose),

**V7.3**—the percentage volume of the hippocampal avoidance area receiving ≥ 7.3 Gy.

These parameters were selected as potential predictors of late radiation-induced cognitive impairment based on previous studies [17]. Notably, a V7.3 < 40% threshold was applied to the hippocampal avoidance area, as proposed by Gu et al., representing one of the few available dose constraints tailored specifically to hippocampal subvolumes in nasopharyngeal cancer radiotherapy [1,18].

No brain-specific dose constraints were used during the original 3D-CRT planning, while QUANTEC guidelines were applied for other critical OARs. The comparative analysis focused on the differences in neural structure dose exposure across the three planning techniques.

### 2.5. Ethical Considerations

This study is part of the RETRO-HN2025 project and was approved by the Ethics Committee of the Regional Institute of Oncology (approval no. 28 of 4 February 2025). All procedures were conducted in compliance with institutional guidelines and the principles of the Declaration of Helsinki.

## 3. Results

The dosimetric evaluation focused on the radiation exposure of the temporal lobes, hippocampi, and hippocampal avoidance areas across the three radiotherapy techniques: 3D conformal radiotherapy (3D-CRT), intensity-modulated radiotherapy (IMRT), and volumetric-modulated arc therapy (VMAT).

### 3.1. Temporal Lobe Dosimetry

As shown in Table 1, the minimum dose (D_min) to the temporal lobes ranged from 41 cGy to 146.7 cGy for the 3D-CRT technique (average: 95.49 cGy), 52.6 cGy to 163.3 cGy for IMRT (average: 101.89 cGy), and 66.9 cGy to 3540 cGy for VMAT (average: 5158.97 cGy).

Maximum dose (D_max) values varied from 1532.2 cGy to 6640.7 cGy (3D-CRT), 1857.6 cGy to 7232.4 cGy (IMRT), and 1481.4 cGy to 7067.5 cGy (VMAT), with respective averages of 5682.09 cGy, 5293.93 cGy, and 5158.97 cGy.

Mean dose (D_mean) values ranged from 235 cGy to 2271.8 cGy for 3D-CRT (average: 933.5 cGy), 189.6 cGy to 1928.7 cGy for IMRT (average: 813.95 cGy), and 277.4 cGy to 2154.9 cGy for VMAT (average: 903.21 cGy).

### 3.2. Hippocampus Dosimetry

The dosimetric parameters for the hippocampus are presented in Table 2. D_min ranged from 113 cGy to 765.8 cGy (3D-CRT, average: 2900.2 cGy), 123.5 cGy to 550.5 cGy (IMRT, average: 271.49 cGy), and 179.9 cGy to 777.9 cGy (VMAT, average: 387 cGy).

D_max values ranged from 409.4 cGy to 6393.9 cGy (3D-CRT, average: 2600.53 cGy), 376.1 cGy to 5157.5 cGy (IMRT, average: 2409.05 cGy), and 567.6 cGy to 5485.7 cGy (VMAT, average: 2481.8 cGy).

D_mean ranged from 247.9 cGy to 1935.2 cGy for 3D-CRT (average: 747.51 cGy), 217.4 cGy to 1860.2 cGy for IMRT (average: 891.48 cGy), and 355.8 cGy to 2505.2 cGy for VMAT (average: 1077.06 cGy).

### 3.3. Hippocampal Avoidance Area Dosimetry

As detailed in Table 3, D_max for the hippocampal avoidance region ranged from 6.44 cGy to 6638.5 cGy (3D-CRT, average: 4201.17 cGy), 640.5 cGy to 7161.8 cGy (IMRT, average: 3889.33 cGy), and 784.7 cGy to 6606.8 cGy (VMAT, average: 3619.91 cGy).

The V7.3 values—indicating the percentage of the hippocampal avoidance volume receiving ≥ 7.3 Gy—varied between 0.0% and 90.81% for 3D-CRT (mean: 28.67%), 0.0% and 88.7% for IMRT (mean: 37.79%), and 0.0% and 96.2% for VMAT (mean: 42.45%).

### 3.4. Summary and Interpretation

Across all three techniques, the average D_max values for both hippocampus and temporal lobes often exceeded recommended safety thresholds when neural structures were not actively spared. Notably, certain VMAT and IMRT plans resulted in temporal lobe D_max values surpassing the suggested clinical threshold of 70 Gy.

While inverse planning techniques (IMRT and VMAT) led to reduced mean doses to the temporal lobes (D_mean), they also increased the D_min values compared to 3D-CRT, possibly due to the broader distribution of low- and moderate-dose regions. For hippocampus and hippocampal avoidance structures, IMRT yielded the lowest median D_max, though at the cost of slightly elevated D_min and D_mean compared to 3D-CRT.

These results emphasize the importance of delineating the hippocampi and temporal lobes as organs at risk (OARs) in radiotherapy planning to mitigate the risk of neurocognitive sequelae. The variability observed among techniques also reinforces the value of individualized treatment planning and the integration of hippocampal-sparing objectives.

### 3.5. Visual Data Summary

**Figure 2:**
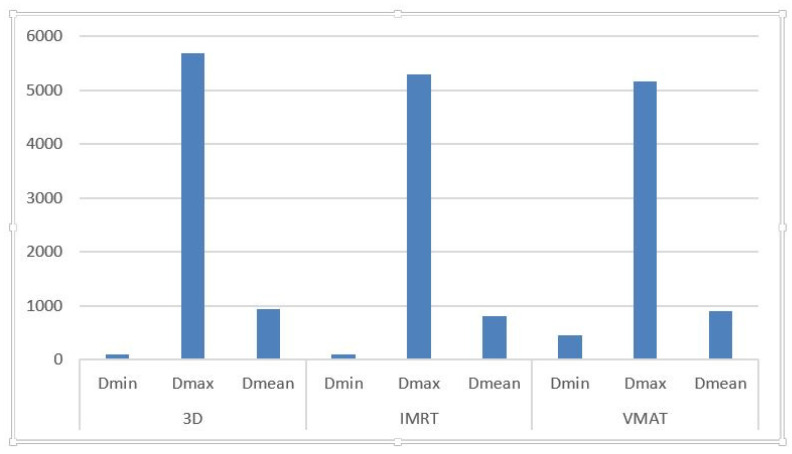
Mean (D_mean), minimum (D_min), and maximum (D_max) radiation doses delivered to the temporal lobes across all three radiotherapy techniques (3D-CRT, IMRT, and VMAT). Data reflect patient-specific outputs from the treatment planning system and highlight technique-dependent variation in neural structure exposure.

Figure 2: Mean (D_mean), minimum (D_min), and maximum (D_max) radiation doses received by the temporal lobes.

**Figure 3:**
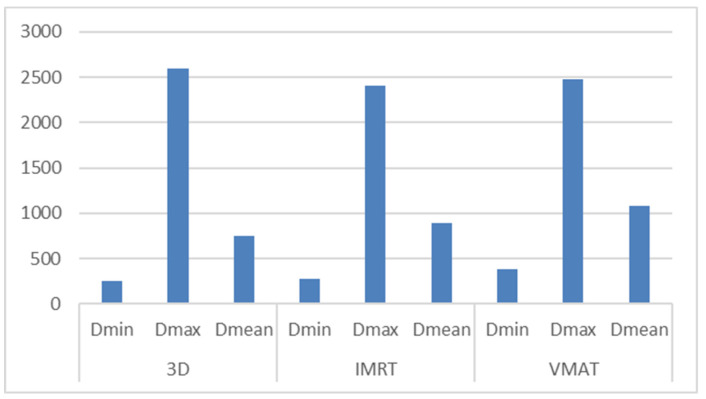
Dosimetric comparison of hippocampal exposure for 3D-CRT, IMRT, and VMAT plans, including D_mean, D_min, and D_max values. The figure illustrates that despite inverse planning techniques reducing D_max in some cases, mean and minimum doses tend to increase compared to 3D-CRT.

Figure 3: Corresponding dose values for the hippocampus.

**Figure 4:**
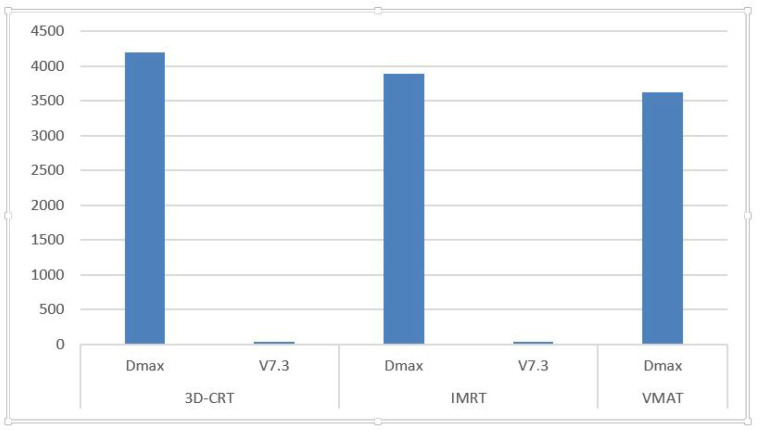
Maximum dose (D_max) and V7.3 values in the hippocampal avoidance area for each treatment technique. V7.3 refers to the percentage of the hippocampal avoidance region receiving ≥7.3 Gy. Several plans exceeded the recommended V7.3 < 40% constraint, indicating potential risk for cognitive impairment.

Figure 4: Maximum dose (D_max) and V7.3 for the hippocampal avoidance area.

All dose values were extracted directly from the treatment planning system (TPS) outputs and reflect real patient plans without prior optimization for neural OARs.

### 3.6. Key Findings

- IMRT and VMAT offer superior target coverage but may increase mean and minimum doses to sensitive neural structures.
- Although median D_max values can be reduced by inverse planning, they may still exceed safe limits if plans are not specifically optimized.
- In several cases, V7.3 values for the hippocampus exceeded the recommended 40% threshold, suggesting elevated risk for neurocognitive decline.
- Temporal lobe D_max values in certain VMAT plans surpassed the suggested 65–70 Gy limit, reinforcing the need for proactive dose constraint application.

## 4. Discussion

### 4.1. If We Use Dose-Volume Recommendations from Whole-Brain Radiotherapy (WBRT)

The hippocampus and the entire limbic system have a demonstrated role in memory. Preclinical studies have correlated stem cell loss, as well as structural and functional changes in mature neurons, with radiation exposure. The hippocampus, composed of the dentate gyrus and cornu ammonis regions, is a central component of the limbic system. The rationale for sparing and reducing the dose to the hippocampus lies in the goal of preserving cognitive function [19].

Based on this preclinical evidence, Phase II trials were initiated to evaluate hippocampal dose-reduction in WBRT using linear accelerator (LINAC)-based intensity-modulated radiation therapy (IMRT) or helical tomotherapy. Normalizing the dose to 2 Gy (prescription: 30 Gy in 10 fractions), the mean hippocampal dose was reduced by 87% and 81%—to 0.49 Gy and 0.73 Gy—using helical tomotherapy and LINAC-based IMRT, respectively.

Applying the linear-quadratic model, the researchers transformed the biologically effective dose (BED) into equivalent doses in 2 Gy fractions (EQD2), correlating radiation dose with cognitive deficits. A higher volume (>40% of the bilateral hippocampus) receiving at least 7.3 Gy was identified in several studies, indicating an increased risk of long-term cognitive impairment [1,6]. This threshold (V7.3 < 40%) is now used in hippocampal-sparing protocols.

In radiotherapy for head and neck cancers, the introduction of IMRT—offering steep dose gradients and high conformality—resulted in unintended exposure of extra-target brain regions. Although IMRT significantly reduced grade 3 or higher toxicities, such as xerostomia and mucositis, and decreased feeding tube dependence [20], its increased “low-dose bath” to surrounding tissues raises concern about delayed cognitive side-effects.

VMAT (volumetric-modulated arc therapy) delivers radiation via continuous gantry rotation, enabling treatment from multiple 360° beam angles [21]. VMAT plans are considered equivalent or superior to step-and-shoot IMRT in terms of target coverage and OAR sparing, with a ~50% reduction in treatment delivery time [22,23].

It is essential to reconcile that IMRT and VMAT, while reducing maximum doses in certain subvolumes, may inadvertently increase exposure in other brain regions due to highly conformal dose gradients. These findings emphasize the importance of meticulous planning and structure delineation.

Our findings reinforce the necessity of implementing hippocampal-sparing protocols in head and neck radiotherapy. Moreover, integrating clinical and genetic predictors—such as polygenic risk scores (PRS)—into personalized planning models could enhance outcomes and reduce the risk of temporal lobe necrosis (TLN) and cognitive impairment.

Hippocampal irradiation tolerance remains poorly defined. Most patients treated for brain metastases or gliomas do not survive long enough to experience cognitive impairment. Moreover, medications and tumor recurrence also contribute to cognitive decline, complicating the isolation of radiotherapy effects [24].

In patients with locally advanced nasopharyngeal cancer (NPC), particularly those with skull base invasion, irradiation of brain structures such as the hippocampus and temporal lobes is associated with neurocognitive decline (NCF). Bilateral hippocampi and temporal lobes were delineated using the RTOG atlas on T1-weighted MRI, co-registered with planning CT via rigid registration [25].

The following radiobiological formulas were considered in the analysis:

**Biologically Effective Dose (BED)** = *n* × *d*(1 + *d*/α/β)

**Equivalent Dose in 2 Gy Fractions (EQD2)** = *D* × [(*d* + α/β)/(2 + α/β)]

where *n* = number of fractions, *d* = dose per fraction (Gy), *D* = total dose (Gy), and α/β = 10 for tumors, 2–3 for late effects [26].

Khodayari et al. assessed hippocampal dose in IMRT for NPC using 70 Gy in 2.12 Gy daily fractions. In 30% of cases, the hippocampus received a higher dose than the target volume. Their findings indicate that, without specific dose constraints, the hippocampus may inadvertently receive high radiation doses during conformal IMRT planning [27].

Tsai et al. identified EQD2 thresholds predictive of verbal memory impairment at various hippocampal subvolumes: <12.6 Gy (0%), <8.81 Gy (10%), <7.45 Gy (50%), and <5.83 Gy (80%) [28]. The concept of hippocampal-sparing via IMRT for brain tumors has shown early benefits for memory preservation in the first three months post-treatment [29].

Chemotherapy also contributes to cognitive dysfunction—commonly referred to as “chemo-brain”. This phenomenon, involving neuronal inflammation and oxidative stress, occurs despite most chemotherapeutic agents not crossing the blood–brain barrier (BBB). Inflammatory cytokines such as TNF and IL-1/6 can cross the BBB, impairing neurogenesis, reducing dendritic cell integrity, and altering neurotransmitter levels. An experiment on mice demonstrated attention deficits after cisplatin administration—the standard agent in nasopharyngeal chemoradiotherapy—with abnormalities in synaptic integrity observed in the prefrontal cortex [30,31].

Radiation-induced neurotoxicity involves demyelination, direct neuronal and vascular injury, inflammation, and BBB permeability changes. Early lesions appear within 1–6 months, while late lesions manifest after six months. Pharmacologic agents under investigation for neuroprotection include Donepezil, Memantine, Methylphenidate, Armodafinil, and Gingko Biloba. Additionally, Metformin and Ramipril have shown promise in preclinical studies for mitigating radiation-related cognitive decline [32,33,34,35,36,37].

Recent studies link high radiation dose to the left hippocampus with impaired verbal memory and fluency. Similarly, doses to the left temporal lobe and thalamus correlate with executive dysfunction and slowed processing speed. These findings highlight the need to identify and protect multiple neurocognitive structures—including the hippocampus, thalamus, and temporal lobes—during curative-intent radiotherapy for NPC [38,39,40].

The current standard for locally advanced NPC includes induction chemotherapy with Gemcitabine-Cisplatin followed by concurrent chemoradiotherapy. Both agents are associated with impaired brain metabolism. Simultaneous integrated boost (SIB) IMRT, delivering > 2 Gy per fraction, such as 2.12 Gy over 33 fractions, may heighten brain toxicity risk [40,41,42,43,44,45,46].

Multi-parametric MRI techniques such as 3D-pCASL and IVIM have enabled early detection of microscopic structural changes in neural tissue following IMRT for NPC. These parameters, along with the perfusion fraction (F) derived from IVIM, were assessed over time. The F-values exhibited an oscillating trend during follow-up. Notable changes include increased cerebral blood flow (CBF) at one week post-treatment, and rising diffusion parameters (D, D*) at six months—consistent with apoptosis and reduced cell density. These imaging biomarkers may offer predictive insight into long-term cognitive effects [47,48].

Early post-radiation microstructural changes have been linked to late neurotoxicity. Monitoring these changes before symptom onset may help prevent irreversible damage [49,50].

He et al. proposed a predictive model incorporating a polygenic risk score (PRS) and clinical factors (age, TNM stage, dose) to stratify TLN risk in 1189 patients. Dose thresholds were 57.16 Gy for low-risk and 68.1 Gy for high-risk PRS groups [51]. Wen et al. demonstrated that adding clinical data (age, stage) to radiobiological models improves TLN prediction at 36 months, with D0.5cc > 65.06 Gy as a key predictor [52].

A comparison of our findings with the existing literature is provided in Table 4. Our results confirm elevated hippocampal and temporal lobe exposure in VMAT plans, with occasional V7.3 values exceeding 40%, aligning with previous data on risk thresholds.

Skull base tumors, including glioblastomas, pose similar challenges due to complex radiation fields. Thus, strategies to protect cognitive function in such contexts also apply to NPC [53].

### 4.2. Limitations and Future Directions

This study was based on a small cohort and utilized retrospective treatment planning data without clinical neurocognitive follow-up. Future research should validate these findings with prospective, longitudinal studies integrating cognitive outcomes, functional imaging, and hippocampal-sparing techniques.

We have carefully curated the literature to maintain objectivity and limit over-reliance on self-citations, ensuring balanced, evidence-based interpretation of the results.

## 5. Conclusions

As survival outcomes continue to improve for patients treated with curative-intent radiotherapy for locally advanced nasopharyngeal cancer, the long-term preservation of cognitive function must be prioritized as a critical component of comprehensive cancer care. The integration of intensity-modulated irradiation techniques and induction chemotherapy followed by concurrent chemoradiotherapy has significantly improved locoregional control, yet also introduces new challenges related to the unintentional exposure of neural structures to radiation.

This study demonstrates that without the inclusion of specific dose constraints, critical regions such as the hippocampus and temporal lobes are at risk of receiving clinically significant radiation doses, particularly with highly conformal techniques like IMRT and VMAT. Our findings underscore the importance of routine delineation of these neural structures as organs at risk (OARs) and the adoption of evidence-based dose thresholds—such as V7.3 < 40% for the hippocampus—derived from whole-brain radiotherapy data and supported by emerging predictive models.

In particular, the risk of elevated D_max in the temporal lobes and hippocampus, especially when not constrained, presents a potential hazard for neurocognitive decline and radiation-induced dementia. The application of biologically based dose modeling (EQD2), advanced imaging biomarkers, and polygenic risk scores offers a promising path toward personalized treatment planning and neuroprotection.

Given the increased therapeutic intensity of modern NPC management, **hippocampal sparing and temporal lobe dose minimization should become standard considerations** in radiotherapy protocols. Dose optimization strategies that incorporate clinical, dosimetric, and genetic predictors can reduce cognitive risks without compromising oncologic efficacy.

We recommend the routine implementation of hippocampal-sparing approaches in the treatment of nasopharyngeal cancer, alongside the delineation of the temporal lobes, as a practical and necessary step to mitigate neurocognitive toxicity in long-term survivors. Future research should build on these findings by incorporating clinical neurocognitive outcomes and expanding cohort sizes to validate thresholds and models in diverse populations.

## Figures and Tables

**Table 1:** Temporal lobe dosimetry parameters across 3D-CRT, IMRT, and VMAT techniques.

Technique	D_Min (cGy)	D_Max (cGy)	D_Mean (cGy)
3D-CRT	41–146.7 (avg: 95.49)	1532.2–6640.7 (avg: 5682.09)	235–2271.8 (avg: 933.5)
IMRT	52.6–163.3 (avg: 101.89)	1857.6–7232.4 (avg: 5293.93)	189.6–1928.7 (avg: 813.95)
VMAT	66.9–3540 (avg: 5158.97)	1481.4–7067.5 (avg: 5158.97)	277.4–2154.9 (avg: 903.21)

**Table 2:** Hippocampus dosimetry across all three radiotherapy techniques.

Technique	D_Min (cGy)	D_Max (cGy)	D_Mean (cGy)
3D-CRT	113–765.8 (avg: 2900.2)	409.4–6393.9 (avg: 2600.53)	247.9–1935.2 (avg: 747.51)
IMRT	123.5–550.5 (avg: 271.49)	376.1–5157.5 (avg: 2409.05)	217.4–1860.2 (avg: 891.48)
VMAT	179.9–777.9 (avg: 387)	567.6–5485.7 (avg: 2481.8)	355.8–2505.2 (avg: 1077.06)

**Table 3:** Dosimetric values for the hippocampal avoidance area, including D_max and V7.3.

Technique	D_Max (cGy)	V7.3 (%)
3D-CRT	6.44–6638.5 (avg: 4201.17)	0.0–90.81 (mean: 28.67)
IMRT	640.5–7161.8 (avg: 3889.33)	0.0–88.7 (mean: 37.79)
VMAT	784.7–6606.8 (avg: 3619.91)	0.0–96.2 (mean: 42.45)

**Table 4:** Comparison of current study findings with key literature.

Study/Source	Population/Setting	Key Findings	Recommendations/Implications
Current Study (3D-CRT vs. IMRT/VMAT for NPC)	Locally advanced NPC; comparative dosimetric study	D_max exceeded constraints in several plans; IMRT/VMAT reduced D_mean but increased D_min in hippocampus; V7.3 often >40% in VMAT plans	Reinforces the need to routinely delineate hippocampus and temporal lobes as OARs; advocates for individualized planning
Gu et al. (VMAT/NPC) [1]	Locally advanced NPC using VMAT	Proposed V7.3 < 40% constraint for hippocampus; higher hippocampal exposure linked to cognitive risk	Advocates for integrating V7.3 as a planning constraint in NPC radiotherapy
Gondi et al. (IMRT/WBRT) [16]	Brain metastases, WBRT hippocampal sparing	87–81% reduction in hippocampal dose with sparing; short-term memory preservation achieved	Supports routine hippocampal sparing to maintain cognitive function
Khodayari et al. (IMRT/NPC) [27]	NPC treated with IMRT	In 30% of plans, hippocampus received a higher dose than tumor; lack of OAR constraint resulted in unintended exposure	Highlights need for hippocampus delineation in head and neck radiotherapy planning
Tsai et al. (WBRT + Sparing) [28]	WBRT with hippocampal sparing	Verbal memory impairment correlated with EQD2 values of hippocampal subvolumes; dose thresholds proposed for memory loss prevention	Supports subvolume-based dose limits for hippocampus to preserve verbal memory

## Data Availability

The original contributions presented in this study are included in the article. Further inquiries can be directed to the corresponding author.
